# Suicidal behavior amongst adolescent students in south Delhi

**DOI:** 10.4103/0019-5545.39756

**Published:** 2008

**Authors:** Rahul Sharma, Vijay L. Grover, Sanjay Chaturvedi

**Affiliations:** Department of Community Medicine, University College of Medical Sciences, New Delhi, India

**Keywords:** Adolescent, students, suicidal behavior, suicide

## Abstract

**Objective:**

To study the prevalence of suicidal behavior and its epidemiological correlates amongst adolescent students in south Delhi.

**Settings and Design:**

A cross-sectional study in three schools and two colleges in south Delhi.

**Participants:**

A total of 550 adolescent students aged 14 to 19 years selected by cluster sampling.

**Statistical Analysis:**

Proportions, chi square test, bivariate logistic regression.

**Results:**

About 15.8% reported having thought of attempting suicide, while 28 (5.1%) had actually attempted suicide, both being more in females than in males. Statistically significant associations were observed with the age of the student, living status of parents, working status of mother, and whether the student was working part-time. The two variables found significant on multivariate analysis were female gender and the number of role models the student had ever seen smoking or drinking.

**Conclusion:**

The prevalence of suicide-risk behavior was found to be quite high and is a matter that should evoke public health concern.

## INTRODUCTION

The World Health Organization has defined “adolescents” as persons in the 10 to 19-year age group.[1] Today India has a population of adolescents that is among the largest in the world. This is the generation which will shape India's future. One of the most important commitments a country can make for its future economic, social, and political progress and stability is to address the health- and development-related needs of its adolescents.[2]

The present study covered six categories of the important health risk behaviors among adolescents. These included tobacco use, alcohol and other drug use, sexual risk behaviors, unhealthy dietary behaviors, inadequate physical activity, and behaviors that may result in injuries and violence. From the review of available literature, it was seen that very few studies have been carried out in India; and in Delhi, in particular, which comprehensively cover the health risk behaviors among the adolescent student population, especially in the recent few years. In this paper, the findings related to suicidal behavior among the adolescent students are presented.

Suicidal behavior amongst adolescent students is a matter of great concern due to the tragic loss of prime years of life it entails. It is vital to study both the prevalence and the correlates of such behaviors.

## MATERIALS AND METHODS

The study was a cross-sectional analysis of the subject population. The units of the study were 14- to 19-year-old adolescents studying in various schools and colleges in south Delhi. The study being a doctoral thesis was reviewed and approved by the institutional ethics committee. For the purpose of the present study, two districts of Delhi, south and southwest districts, were together considered as south Delhi region. All the schools and colleges in south Delhi region were included in the sampling frame. A two-stage cluster sampling design was used to draw a representative sample of students in classes 9 to 12 in schools and the first two years of graduation in colleges. These classes were chosen as they correspond to the desired age group of 14 to 19 years. Details of the methodology have been published earlier.[3]

All students from the selected classes present on the day of the survey were eligible to participate, allowing for anonymous and voluntary participation. At the time of data analysis, the forms of respondents who had stated their age to be either less than 14 years or more than 19 years were excluded from the analysis. A pre-tested, semi-open-ended and self-administered questionnaire was used in the study. Statistical analysis of the data was done on the SPSS software, using cross-tabulation with the chi-square test. Binary logistic regression was applied to analyze the relationship between suicidal risk behavior and various independent variables under study.

## RESULTS

The mean age of the respondents was 16.5 ± 1.5 years. Overall, among the 550 respondents, there were 67.1% males and 32.9% females. Large majority of the respondents were Hindus (492, or 89.6%). A majority of subjects (343, or 62.4%) reported their place of residence as being a private colony or a separate bungalow. Three-fourths (407) belonged to a nuclear family, and the remaining were a part of a joint family. Among the students, 40 (7.3%) reported doing some part-time work for income after school/college hours. Of these, 28 were males while 12 were females. More collegians (9.6%) than school students (6.4%) were working for income. Majority of the subjects who reported working gained income by giving tuitions. The students were asked to mention if they had ever seen any of the six “role models” smoking cigarettes and/or drinking alcohol, which included father, mother, sibling, best friend, favorite teacher, and favorite celebrity.

The study included two questions apropos suicidal behavior: about seriously contemplating suicide and about actually attempting suicide. The period under consideration for both the queries was past 12 months. The results are depicted in Table 1. Suicidal thoughts and having attempted suicide were found to be more prevalent in females than in males. One in every five female students (19.9%) reported having seriously considered suicide, while 13 (7.2%) went ahead and actually attempted suicide. Another matter of concern was that 5 (1.4%) males and 6 (3.4%) females reported having made multiple attempts at committing suicide within the past 12 months.

**Table 1:** Suicide-risk behaviors among the adolescent students

Behavior	Males (*n* = 369)		Females (*n* = 181)		Total (*n* = 550)	
			
	*n*	%	*n*	%	*n*	%
Seriously considered attempting suicide in past 12 months	51	13.8	36	19.9	87	15.8
Actually attempted suicide ≥ 1 time in past 12 months	15	4.1	13	7.2	28	5.1

The association of suicide-risk behavior with a range of socio-demographic factors concerning adolescents was explored as part of the study. The significant results are presented in Table 2. The suicide-risk behavior was more among the higher-age groups (about 18% in each) than among the age group of 14 to 15 years (10.3%) [*P* = 0.075]. Statistically significant associations were observed with living status of the parents and with working status of the subject. Pupils with working mothers were significantly more likely to be at risk than were those whose mothers were homemakers. A significant association (*P* < 0.001) was observed with the number of role models ever seen smoking or drinking.

**Table 2:** Association of suicide-risk behavior with various socio-demographic variables

Study characteristic	Total number of respondents	Number having ‘at risk’ behavior	%	*P* value
Age (in years)				
14-15	155	16	10.3	0.075
16-17	238	43	18.1	
18-19	157	29	18.5	
Total	550	88	16.0	
Gender				
Male	369	52	14.1	0.08
Female	181	36	19.9	
Total	550	88	16.0	
Status of parents				
Both alive	511	77	15.1	0.035
One parent not alive	22	7	31.8	
Total	533	84	15.8	
Respondent working for income after school/college				
Yes	40	11	27.5	0.041
No	507	77	15.2	
Total	547	88	16.1	
Working status of mother				
Home-maker	430	59	13.7	0.018
Working	109	25	22.9	
Total	539	84	15.6	
Role models seen smoking or drinking (out of six asked about)				
None	131	10	7.6	<0.001
1-2	353	58	16.4	
Three or more	66	20	30.3	
Total	550	88	16.0	

The total for some of the study characteristics is less than 550 due to missing responses

Table 3 shows the significant findings on binary logistic regression analysis that adjusted for various variables. Female gender was found to be strongly associated with suicidal behaviors. Another significant correlate was the number of role models ever seen smoking or drinking. Compared to those who had never seen any role model smoke/drink, those who had seen one or two role models smoke/drink were twice as likely; and those who had seen three or more were five times as likely to be indulging in suicide-risk behavior.

**Table 3:** Binary logistic regression analysis for significant correlates of suicide-risk behavior

S. no.	Correlates	Categories	Adjusted odds ratio (95% CI)	*P* value
1	Gender	Male	1 (Reference)	-
		Female	1.934 (1.151-3.248)	0.013
2	Role models seen smoking or drinking	None	1 (Reference)	-
		1-2	2.095 (1.025-4.281)	0.042
		3 or more	5.345 (2.213-12.909)	<0.001

## DISCUSSION

Health-risk behaviors are an important link to the overall health status of adolescents and are important determinants of morbidity and mortality in this age group.[4] In the present study, females were found to be more likely to report suicidal tendencies than males (OR: 1.934; 95% CI: 1.151-3.248). This result is in concurrence with international experience.[4–8] The same finding was also observed in the Indian studies by Logaraj *et al.* and by Lalwani *et al.*[9,10]

Bearman *et al.* mention that it is expected that suicidal ideation is about three times more prevalent compared to actual suicide attempts.[11] This was confirmed by the findings from the present study. Previous studies too have found a similar range for the prevalence of suicidal ideations.[5,7,12] Having seriously considered and/or actually attempted suicide within the past 12 months was more among the higher-age groups. It has been mentioned that suicide is rare before puberty, but the rate begins to rise sharply after the age of 14 years. Adolescents in the late stage of adolescence are likely to be experiencing more stress and emotional turmoil as they face the threshold of adulthood. In this period, rising expectations and responsibilities may create pressures for many of them.

The findings that students with a parent not alive and those whose mothers were working were at a higher risk regarding suicidal behaviors, suggest the important role that parental support and availability plays in ensuring the adolescents’ capacity to prevail over various stresses in their lives. Murphey *et al.*[13] too had found communication with parents to be a vital youth “asset” against different health-risk behaviors, including suicidal thoughts.

A substantial association was observed with the number of role models ever seen smoking or drinking, for suicide-risk behavior. The relationship between having seen important people in their lives smoke or drink and indulging in different negative behaviors that are manifestly detrimental to health, would be difficult to justify as being direct or causal. However, seeing one or more of role models in their environment resorting to any one risk behavior, smoking or drinking, may develop a feeling of acceptance of these in their social circle. This removes their inhibitions and allows them to adopt these and other health-risk behaviors.

A point to be kept in mind is that the findings of the present study and their interpretations are restricted to adolescent students only. Further studies are needed that cover the group of out-of-school adolescents too, to find the extent of risk behaviors and means to tackle such behaviors among these subsets of the adolescent population. The patterns of suicide-risk behavior found among the adolescent students in our study were consistent with those seen among the adolescents around the world. Considering the large number of adolescents in our country, the study thus evokes an urgent need to stimulate an action to identify those at risk and those who need treatment among the adolescent student populace. The results of this assessment would have a direct influence on how programs and approaches may best be framed and put into practice to deal with risk behaviors in this vital subset of the population.
